# Multiple Pyogenic Arthritis Caused by *Edwardsiella tarda* Bacteremia: A Case Report and Literature Review

**DOI:** 10.1155/crdi/6304698

**Published:** 2025-11-14

**Authors:** Ryuto Yokoyama, Kenya Yarimizu, Yu Onodera

**Affiliations:** ^1^Department of Emergency and Critical Care Medicine, Yamagata University Hospital, Yamagata, Japan; ^2^Department of Anesthesiology, Yamagata University Hospital, Yamagata, Japan; ^3^Department of Critical Care, Yonemori Hospital, Kagoshima, Japan

**Keywords:** bacteremia, *Edwardsiella tarda*, infectious disease, pyogenic arthritis, sepsis

## Abstract

Infection with *Edwardsiella tarda*, a Gram-negative rod found in freshwater and seawater, is typically associated with the ingestion of raw fish and commonly causes enteritis, liver abscess, cholangitis, and cholecystitis in humans. However, multiple pyogenic arthritis, which have not been previously reported, are considered exceedingly rare. Herein, we report a case of *E. tarda* bacteremia that resulted in multiple pyogenic arthritis and septic shock, which was successfully treated with intensive care. An 84-year-old man with a history of prostate cancer, diabetes mellitus, and chronic kidney disease was transferred to our hospital for swelling and pain in the left shoulder, following initial treatment for pyogenic arthritis of the left hip at a previous hospital. Imaging revealed the formation of abscesses in the shoulders and hips. He underwent emergent debridement of the left shoulder abscess and was admitted to the ICU. *E. tarda* was identified from blood cultures on Day 3 of hospitalization. The patient developed septic shock and required vasopressor support. Intensive care, including the administration of appropriate antibiotics and surgical debridement, yielded clinical improvement. The patient was discharged from the ICU on Day 8. Cases of multiple pyogenic arthritis caused by *E. tarda* bacteremia have the potential for rapid clinical deterioration. Our patient had multiple risk factors, including cancer, diabetes, and hypertension, which might have predisposed him to a severe disease course. Early diagnosis, appropriate antibiotic therapy, surgical debridement, and intensive care are crucial for patient survival, and early transfer to a tertiary center may improve outcomes. Even rare infections, such as *E. tarda*, should be considered in the differential diagnosis of immunocompromised patients.

## 1. Introduction

*Edwardsiella tarda* is a Gram-negative bacillus found in freshwater and seawater. Infection with *E. tarda*, typically associated with the ingestion of raw fish [1], most commonly causes enteritis, liver abscess, cholangitis, and cholecystitis in humans [2, 3]. The most common underlying disease leading to bacteremia is cancer, accounting for 65% of the cases; patients with immunosuppression, such as those with cancer, are highly likely to develop severe complications [3]. Although bacteremia itself is rare, the mortality rate can be as high as 40% [4]. Previous reports have described monoarticular septic arthritis caused by *E. tarda*, but polyarticular involvement has rarely been documented. The multiplicity of joint involvement in the present case may be attributed to sustained bacteremia and hematogenous dissemination, possibly facilitated by impaired host immunity. This case highlights the potential for *E. tarda* infection to cause disseminated musculoskeletal complications even in the absence of external trauma. Herein, we report a case of *E. tarda* bacteremia that resulted in multiple pyogenic arthritis and septic shock, which was successfully treated with intensive care.

## 2. Case Presentation

An 84-year-old man presented with the chief complaint of bilateral shoulder pain. His medical history included prostate cancer, diabetes mellitus, hypertension, chronic kidney disease, osteoarthritis of the hip, and left ureteral calculus. Additionally, the patient had been hospitalized 4 months prior to the current admission for terminal ileitis. He had been prescribed azilsartan/amlodipine, tramadol, empagliflozin, mecobalamin, limaprost, and loxoprofen. The patients had no known allergies.

Three days before admission, the patient fell at home and developed left hip pain. He was diagnosed with pyogenic arthritis of the left hip at another hospital and underwent femoral head resection and debridement with cefazolin therapy. The following day, the patient developed swelling and pain in the left shoulder, and approximately 100 mL of purulent fluid was aspirated.

Since the previous facility was unable to manage the condition, he was transferred to our tertiary care hospital.

On arrival, his Japan Coma Scale score was 2, Glasgow Coma Scale score was E4V4M6, respiratory rate was 18 breaths/min, heart rate was 86 bpm, blood pressure was 115/61 mmHg, temperature was 37.5°C, and peripheral oxygen saturation was 96% (room air). Physical examination revealed bilateral shoulder swelling and pain with a limited range of motion in the left shoulder.

**Figure 1:**
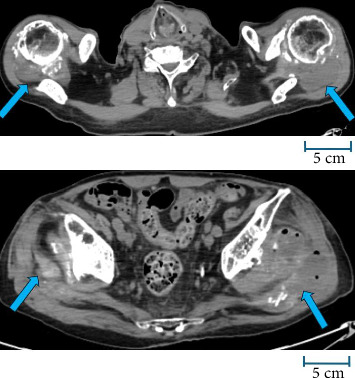
Simple CT scan of the trunk. The blue arrow indicates abscess formation. The scale bar represents a length of 5 cm to reflect the correct physical dimensions.

Laboratory testing results showed elevated C-reactive protein, procalcitonin, blood urea nitrogen, creatinine, and D-dimer and decreased levels of hemoglobin, platelets, and calcium (Table 1). Positive blood cultures for *E. tarda* were obtained in two of two sets (Table 2). Computed tomography revealed abscesses in both shoulders and hips (Figure 1).

**Figure 2:**
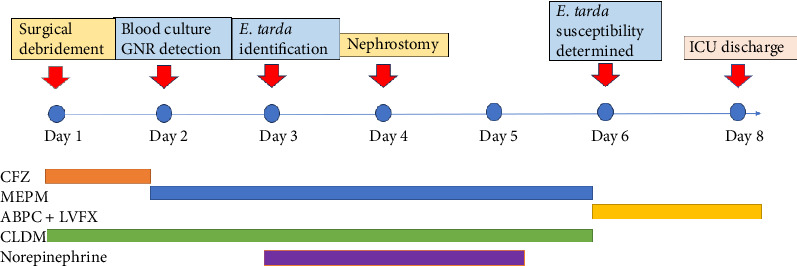
Course of treatment in the ICU.

Following admission, debridement of the left shoulder was performed, and the patient was admitted to the intensive care unit (ICU) for a local gentamicin infusion. On hospital Day 2, Gram-negative rods were identified in blood cultures, prompting an antibiotic change to meropenem. On Day 3, the organism was confirmed to be *E. tarda*, and the patient was diagnosed with multiple pyogenic arthritis due to *E. tarda* bacteremia. Circulatory instability developed on the same day, with the systolic blood pressure dropping to 50 mmHg, necessitating norepinephrine administration. Echocardiography performed on Day 4 revealed no evidence of infective endocarditis. On Day 4, oliguria developed, and a bedside ultrasound examination by a urology specialist revealed left hydronephrosis (Grade 3). Left percutaneous nephrostomy was performed due to suspected ureteral obstruction caused by a left ureteral stone. There was no evidence of urinary tract infection. The hemodynamic status stabilized on Day 5. On Day 6, antimicrobial susceptibility to *E. tarda* was confirmed. Consequently, the antimicrobial therapy was modified to ampicillin and levofloxacin, which were empirically selected based on the clinical judgment of the multidisciplinary team at that time. On Day 8, the patient was discharged from the ICU. Repeated blood cultures on Day 6 yielded negative results (Figure 2).

## 3. Discussion

In the present case, the patient was considered to have developed multiple pyogenic arthritides as a result of *E. tarda* bacteremia. A PubMed search using the terms “Edwardsiella tarda” [MeSH] OR “Edwardsiella tarda” [tiab] yielded 345 articles published in the past 5 years. Most of the existing reports describe hepatobiliary infections as the primary manifestation of *E. tarda* infection [5–7]. In contrast, musculoskeletal infections are rarely reported, although a few recent cases of vertebral osteomyelitis have been documented [8]. While rare manifestations of *E. tarda* bacteremia, such as necrotizing fasciitis, meningitis, intra-abdominal abscess, empyema, and infective endocarditis, have been described [9–13], to our knowledge, there have been no reports of multiple pyogenic arthritis. Thus, this case represents a significant contribution to the accumulation of knowledge regarding *E. tarda* bacteremia. The incidence of *E. tarda* bacteremia has been reported to be 0.004% (38/9368), which is extremely low [3]. Among the diseases caused by *E. tarda* bacteremia, hepatobiliary infections account for 84.8% (32/38). Malignancy was the most common underlying condition, present in 65.8% (25/38) of the cases, 23.7% (9/38) of which progressed to sepsis. The all-cause mortality rate was 8.6% (3/35) at 30 days and 25.8% (8/31) at 90 days. A separate study involving 182,668 sets of blood cultures also demonstrated similar findings, with hepatobiliary infections being the predominant clinical presentation and malignancy the most frequent comorbidity [14]. In the present case, the patient had a history of malignancy and diabetes mellitus, suggesting a potentially increased susceptibility to *E. tarda* bacteremia.

We reviewed 12 reported cases of septic shock caused by *E. tarda* infection over the past 15 years (Table 3). The most common underlying disease was liver abscess (33.3%, 4/12), followed by soft tissue infections or necrotizing fasciitis (25.0%, 3/12), and biliary tract infections (16.6%, 2/12). The mortality rate was 33.3% (4/12), with necrotizing fasciitis accounting for 50% (2/4) of the fatal cases. Carbapenem antibiotics were selected as the initial antimicrobial therapy in 83.3% (10/12) of the cases, and source control by drainage was performed in 66.6% (8/12). These findings suggest that the mortality rate in patients who develop septic shock may be higher than that in patients with bacteremia alone. Among the four fatal cases, only one patient—who underwent surgical debridement—survived, while drainage was not performed in the remaining three, who had pneumonia or soft tissue infections. Comparing the drainage group and nondrainage group, the survival rate was 87.5% (7/8) in the drainage group and 25.0% (1/4) in the nondrainage group. Fisher's exact test showed a tendency toward higher survival in the drainage group, but no significant difference was observed (*p* = 0.0667). The hazard ratio for survival was 3.50 (95% CI: 0.63–19.5), and the odds ratio was 21.0 (95% CI: 0.96–456.7). Although statistical significance was not demonstrated, there is potential for improved survival rates. These observations underscore the crucial role of source control in managing septic shock. In the present case, early surgical debridement was performed for pyogenic arthritis, and early diagnosis and timely surgical intervention are likely to have contributed to the patient's survival.

*E. tarda* has been suggested to exist in a state of asymptomatic infection [15]. In general, patients with cancer are at an increased risk for asymptomatic infection or reactivation of pathogens, such as *Mycobacterium tuberculosis* and other bacteria [16, 17]. In the present case, reactivation from a latent *E. tarda* infection might have occurred. The patient had been hospitalized 4 months prior to the current admission for terminal ileitis. At that time, although C-reactive protein levels were markedly elevated (25 mg/dL), the clinical symptoms and imaging findings of enteritis were mild, and the patient was treated with a 5-day course of intravenous cefazolin before being discharged. Given the patient's history of prostate cancer and diabetes mellitus, he was likely in an immunocompromised state. Although stool culture was not performed in this case, *E. tarda* has been reported to be isolated from 0% to 0.8% of clinical stool specimens [18]. Although persistent colonization is uncommon, these findings suggest that *E. tarda* may be transiently carried in the intestinal tract. Therefore, the possibility remains that *E. tarda* persisted as a subclinical infection due to insufficient antimicrobial therapy. In immunocompromised individuals, such as those with malignancy or diabetes, *E. tarda* should be considered a potential pathogen in cases of enteritis, and an adequate duration of antimicrobial therapy may be warranted.

## 4. Conclusions

Multiple pyogenic arthritis caused by *E. tarda* bacteremia is extremely rare and scarcely reported, yet it has the potential for rapid clinical deterioration. Our patient had multiple risk factors, including cancer, diabetes, and hypertension, which might have predisposed him to a severe disease course. Early diagnosis, appropriate antibiotic therapy, surgical debridement, and intensive care are crucial for patient survival. Additionally, early transfer to a tertiary center can also improve outcomes. Even rare infections, such as *E. tarda*, should be considered in the differential diagnosis of immunocompromised patients.

## Figures and Tables

**Table 1:** Laboratory findings on admission.

		Reference range
*Blood cell counts*		
White blood cells	9.4 × 10^3^/μL	4.0–10 × 10^3^/μL
Red blood cells	4.1 × 10^6^/μL	4.5–5.9 × 10^6^/μL
Hemoglobin	11.1 g/dL	13.5–17.5 g/dL
Platelets	1.1 × 10^6^/μL	1.5–4.0 × 10^6^/μL

*Blood chemistries*		
C-reactive protein	35.8 mg/dL	< 0.3 mg/dL
Calcium	8.2 mg/dL	8.5–10.5 mg/dL
Phosphorus	3.7 mg/dL	2.5–4.5 mg/dL
Sodium	143 mmol/L	135–145 mmol/L
Potassium	4.1 mmol/L	3.5–5.0 mmol/L
Chloride	113 mmol/L	98–106 mmol/L
Procalcitonin	11.8 ng/mL	< 0.05 ng/mL
Aspartate aminotransferase	42 U/L	10–40 U/L
Alanine aminotransferase	12 U/L	5–45 U/L
Total bilirubin	0.6 mg/dL	0.2–1.1 mg/dL
Creatine kinase	275 IU/L	55–170 IU/L
Lactate dehydrogenase	246 U/L	120–240 U/L
Blood urea nitrogen	56 mg/dL	8–20 mg/dL
Creatinine	1.6 mg/dL	0.7–1.1 mg/dL

*Blood coagulation*		
Prothrombin time	13%	10%–13%
Prothrombin time-INR	1.1	0.9–1.1
Activated partial thromboplastin time	27.1 s	25–35 s
D-dimer	3.6 μg/mL	< 0.5 μg/mL

**Table 2:** Antibiotic susceptibility test results.

Antibiotic (formal name)	MIC (μg/mL)	Susceptibility
Ampicillin	≤ 8.0	S
Piperacillin	≤ 8.0	S
Piperacillin/Tazobactam	≤ 16.0	S
Ampicillin/Sulbactam	≤ 8.0	S
Amoxicillin/Clavulanic acid	≤ 8.0	S
Cefazolin	≤ 8.0	S
Cefaclor	≤ 8.0	S
Cefotiam	≤ 8.0	S
Cefmetazole	≤ 16.0	S
Flomoxef	≤ 8.0	S
Cefotaxime	≤ 1.0	S
Ceftazidime	≤ 1.0	S
Ceftriaxone	≤ 1.0	S
Cefoperazone/Sulbactam	≤ 16.0	S
Cefcapene pivoxil	≤ 1.0	S
Cefditoren pivoxil	≤ 1.0	S
Cefepime	≤ 2.0	S
Cefoperazone	≤ 8.0	S
Aztreonam	≤ 4.0	S
Imipenem/Cilastatin	≤ 1.0	S
Meropenem	≤ 0.1	S
Doripenem	≤ 1.0	S
Trimethoprim/Sulfamethoxazole	≤ 2.0	S
Gentamycin	≤ 4.0	S
Tobramycin	≤ 4.0	S
Amikacin	≤ 16.0	S
Minocycline	1.0	I
Ciprofloxacin	≤ 0.2	S
Levofloxacin	≤ 0.1	S
Clindamycin	> 4.0	R
Fosfomycin	≤ 4.0	S
Cefpodoxime proxetil	≤ 2.0	S
Latamoxef	≤ 8.0	S
Ceftolozane/Tazobactam	≤ 2.0	S

**Table 3:** Literature review of septic shock caused by *Edwardsiella tarda* infection.

**Basic information**						
**Year**	**Author**	**Infection site**	**Age**	**Country**	**Journal**	

2025	Yan Zhou et al.	Acute gastroenteritis	32F	China	BMC Infectious Diseases	
2025	Fujita M et al.	Pneumonia	90M	Japan	Journal of Infection and Chemotherapy	
2024	Hasegawa M et al.	Recurrent cholangitis and bacteremia	82M	Japan	Oxford Medical Case Reports	
2024	Hiroki Ueda et al.	Fulminant necrotizing fasciitis	58M	Japan	Journal of Infection and Chemotherapy	
2022	Yue Ding et al.	Acute cholangitis	64M	China	Ann Clin Microbiol Antimicrob.	
2022	Ryutaro Tominaga et al.	Emphysematous liver abscess	51F	Japan	IDCases.	
2021	Kevin D. Healey et al.	Sepsis with shock	59F	USA	Am J Case Rep.	
2020	Alireza Hamidian Jahromi et al.	Soft tissue infections	58M	USA	Plast Reconstr Surg Glob Open.	
2020	Gultakin Hasan Bakirova et al.	Multiple liver abscesses	37F	Saudi Arabia	J Med Case Rep.	
2012	Anulekha Mary John et al.	Multiple liver abscesses	18M	India	Indian J Med Microbiol	
2019	Tadashi Yamamuro et al.	Necrotizing fasciitis in both legs	64F	Japan	J Infect Chemother	
2012	Yoshiko Ohara et al.	Liver abscess	85F	Japan	Intern Med	

**Clinical course and treatment**						
**Year**	**Author**	**Time to death**	**Initial antibiotics**	**Postculture antibiotics**	**Past history**	**Treatment**

2025	Yan Zhou et al.	Survived	Imipenem, vancomycin	Piperacillin–tazobactam	Systemic lupus erythematosus, rheumatoid arthritis	None
2025	Fujita M et al.	18 h	Meropenem, levofloxacin	—	Hypertension, hyperuricemia, epilepsy, gastric ulcer, spinal stenosis	None
2024	Hasegawa M et al.	Survived	Meropenem	Meropenem	Cholangitis, bile duct stent, atrial fibrillation, CHF, HTN, CKD, hyperuricemia	Biliary stent
2024	Hiroki Ueda et al.	∼5 h after surgery	Vancomycin, clindamycin	—	Alcoholic liver cirrhosis	Debridement
2022	Yue Ding et al.	Survived	Imipenem–cilastatin	Cefoperazone/sulbactam	Hepatitis B virus infection	Bile resection, stone drainage, T-tube drainage, cholecystectomy
2022	Ryutaro Tominaga et al.	Survived	Meropenem	Ampicillin/sulbactam	SLE, antiphospholipid syndrome, diabetes, choledocholithiasis	Percutaneous transhepatic abscess drainage
2021	Kevin D. Healey et al.	∼34 h later	Cefepime, metronidazole, levofloxacin	—	Lung cancer, PH, liver cirrhosis, hepatitis C, alcohol abuse	None
2020	Alireza Hamidian Jahromi et al.	5 days	Not specified	Not specified	Laparoscopic cholecystectomy 10 months earlier	None
2020	Gultakin Hasan Bakirova et al.	Survived	Piperacillin–tazobactam ⟶ Meropenem + Linezolid	Ciprofloxacin + gentamicin	Alcohol abuse, hepatitis C	Percutaneous drainage
2012	Anulekha Mary John et al.	Survived	Meropenem + Metronidazole	Not specified	Cushing's syndrome	Abscess drainage
2019	Tadashi Yamamuro et al.	Survived	Meropenem, vancomycin, ciprofloxacin	Cefazolin	None	Surgical debridement
2012	Yoshiko Ohara et al.	Survived	Meropenem	Meropenem	Thyroid cancer, diabetes, hypertension	Abscess drainage

## Data Availability

The data that support the findings of this study are available on request from the corresponding author and are not publicly available due to privacy or ethical restrictions.
